# Silybin Meglumine Mitigates CCl_4_-Induced Liver Fibrosis and Bile Acid Metabolism Alterations

**DOI:** 10.3390/metabo14100556

**Published:** 2024-10-17

**Authors:** Xiaoxin Liu, Ninglin Xia, Qinwei Yu, Ming Jin, Zifan Wang, Xue Fan, Wen Zhao, Anqin Li, Zhenzhou Jiang, Luyong Zhang

**Affiliations:** 1 New Drug Screening and Pharmacodynamics Evaluation Center, State Key Laboratory of Natural Medicines, China Pharmaceutical University, Nanjing 210009, China; liuxiaoxincpu@stu.cpu.edu.cn (X.L.); 3222071652@stu.cpu.edu.cn (N.X.); yuqinwei7213@cpu.edu.cn (Q.Y.); jinming721@stu.cpu.edu.cn (M.J.); 3321071743@stu.cpu.edu.cn (Z.W.); 3124074332@stu.cpu.edu.cn (X.F.); 3321071772@stu.cpu.edu.cn (W.Z.); 3221071643@stu.cpu.edu.cn (A.L.); 2 Center for Drug Research and Development, Guangdong Pharmaceutical University, Guangzhou 510006, China

**Keywords:** silybin meglumine, liver fibrosis, bile acid, carbon tetrachloride, bioinformatics

## Abstract

Background: Altered patterns of bile acids (BAs) are frequently present in liver fibrosis, and BAs function as signaling molecules to initiate inflammatory responses. Silybin meglumine (SLB-M) is widely used in treating various liver diseases including liver fibrosis. However, research on its effects on bile acid (BA) metabolism is limited. This study investigated the therapeutic effects of SLB-M on liver fibrosis and BA metabolism in a CCl_4_-induced murine model. Methods: A murine liver fibrosis model was induced by CCl4. Fibrosis was evaluated using HE, picrosirius red, and Masson’s trichrome staining. Liver function was assessed by serum and hepatic biochemical markers. Bile acid (BA) metabolism was analyzed using LC-MS/MS. Bioinformatics analyses, including PPI network, GO, and KEGG pathway analyses, were employed to explore molecular mechanisms. Gene expression alterations in liver tissue were examined via qRT-PCR. Results: SLB-M treatment resulted in significant histological improvements in liver tissue, reducing collagen deposition and restoring liver architecture. Biochemically, SLB-M not only normalized serum liver enzyme levels (ALT, AST, TBA, and GGT) but also mitigated disruptions in both systemic and hepatic BA metabolism by increased unconjugated BAs like cholic acid and chenodeoxycholic acid but decreased conjugated BAs including taurocholic acid and taurodeoxycholic acid, compared to that in CCl_4_-induced murine model. Notably, SLB-M efficiently improved the imbalance of BA homeostasis in liver caused by CCl_4_ via activating Farnesoid X receptor. Conclusions: These findings underscore SLB-M decreased inflammatory response, reconstructed BA homeostasis possibly by regulating key pathways, and gene expressions in BA metabolism.

## 1. Introduction

Liver fibrosis is a pathological condition characterized by the excessive accumulation of fibrous connective tissue in the liver. It typically arises from chronic liver injury and inflammation. Common causes of liver fibrosis include chronic viral hepatitis infections (such as hepatitis B and C), alcohol abuse, non-alcoholic fatty liver disease, and genetic disorders [1,2]. Liver fibrosis may lead to cirrhosis, which is characterized by severe and irreversible scarring of the liver [3]. Cirrhosis can further progress to complications like liver failure and an increased risk of liver cancer [4,5]. Liver fibrosis management involves treating underlying causes, lifestyle changes, medications to reduce inflammation and fibrosis, antioxidant therapy, and regular monitoring of liver function and fibrosis progression [6]. Current treatments for liver fibrosis include agents like belapectin, cenicriviroc, and obeticholic acid, targeting various fibrosis pathways [7]. However, the development of effective liver fibrosis drugs is challenging, with many candidates showing limited success in clinical trials [7]. Thus, there is a pressing need to develop more effective and specifically targeted therapies for liver fibrosis.

Bile acids (BAs) are amphipathic molecules synthesized in hepatocytes from cholesterol, playing a pivotal role in fat digestion [8]. BAs can be classified into unconjugated and conjugated forms, with unconjugated BAs free of amino acid conjugation, while conjugated BAs are primarily bound to glycine or taurine [9]. Unconjugated BAs primarily facilitate fat digestion and absorption, while conjugated BAs enhance solubility and participate in dietary fat emulsification and absorption [10]. BA dysregulation plays a critical role in the pathogenesis of liver fibrosis, potentially acting as both a critical indicator and an etiologic factor. BAs have been found to exert pro-fibrogenic effects on hepatic stellate cells (HSCs), contributing to fibrosis progression [11,12,13]. During the continuous liver injury and repair induced by chronic inflammation, the metabolic network of the liver changes, affecting multiple endogenous metabolites and their associated pathways. This exacerbates liver fibrosis, creating a vicious cycle of injury and repair [14]. Targeting bile metabolism has emerged as a potential strategy for addressing liver fibrosis [15]. However, further research and clinical studies are needed to fully understand the efficacy and safety of targeting bile metabolism as a therapeutic strategy for liver fibrosis.

Silybin, derived from the milk thistle plant (*Silybum marianum*), has been recognized for its hepatoprotective properties and has been used to treat hepatic fibrosis in clinical settings [16,17,18]. Research suggests that silybin may influence BA metabolism by promoting the secretion of BAs into the bile ducts and increasing their excretion from the liver [19]. Additionally, silybin has been investigated for its potential to reduce liver inflammation and oxidative stress, which can indirectly affect BA metabolism [20]. Silybin meglumine (SLB-M), a meglumine salt of silybin has shown increased solubility and minimal cytotoxicity, being widely used in fibrotic treatment in many countries include China. However, the impact and mechanism of silybin meglumine on a BA profile in fibrotic models remain unclear.

This study aimed to deepen our understanding of SLB-M’s therapeutic effects in liver fibrosis using a well-established carbon tetrachloride (CCl_4_)-induced liver fibrosis model in mice [21,22]. Our research focused on evaluating the histopathological, biochemical, and molecular changes induced by SLB-M treatment, with a particular emphasis on its effects on BA metabolism and fibrosis-related gene expression. Our findings may elucidate a new theoretical basis for the treatment of fibrosis with SLB-M.

## 2. Materials and Methods

### 2.1. Animals and Sample Collection

**Figure 1:**
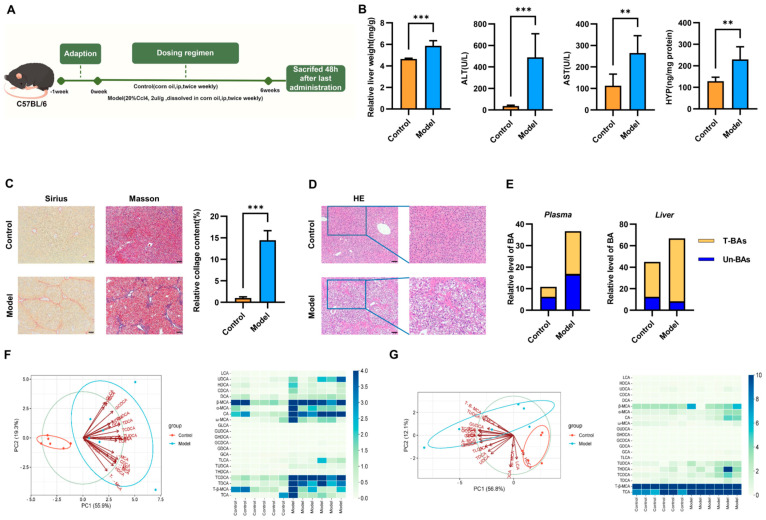
Carbon tetrachloride (CCL_4_)-induced liver fibrosis and bile acid profile alterations in a mouse model. (**A**) A schematic overview of the murine model (by Figdraw 2.0, Hangzhou, China). (**B**) Graphical representation of liver weight and serum markers of liver function. (**C**) Comparative histological outcomes, picrosirius red (**left**) and Masson’s trichrome (**right**), to visualize collagen presence and fibrosis. Scale bar = 100 µm. Relative collagen content of Masson staining. Data represent at least three independent experiments with triplicate measurements. (**D**) Liver sections were stained with hematoxylin and eosin (HE) to visualize structural changes. Scale bar = 100 µm. (**E**) The concentrations of unconjugated BAs and glycine (G)-conjugated and taurine (T)-conjugated BAs in blood and liver samples. (**F**) Principal component analysis (PCA) plots illustrate the distinct metabolic profiles of BAs between the control and model groups in blood and quantification of individual BAs in blood by LC-MS/MS. (**G**) Principal component analysis (PCA) plots illustrate the distinct metabolic profiles of BAs between the control and model groups in liver sample and quantification of individual BAs in liver by LC-MS/MS. Data are expressed as mean ± SEM; ** *p* < 0.01, *** *p* < 0.001 vs. control *n* = 6; HYP: hydroxyproline; ALT: alanine aminotransferase, AST: aspartate aminotransferase.

Male C57BL/6 mice (18–20 g, 6–8 weeks) were purchased from Vital River Laboratory Animal Technology (Shanghai, China). The study was carried out in accordance with the Animal Usage Guidelines by the Animal Ethics Committee of our university (IACUC approval number 2024-07-037). To induce liver fibrosis, the mice underwent a 7-day adaptation period, followed by a 6-week regimen in which they received twice-weekly administrations of 20% CCl_4_ dissolved in corn oil at a dosage of 2 µL/g body weight via intraperitoneal injection [23]. The control group was administered corn oil (Figure 1A). To investigate the therapeutic effects of silybin meglumine on liver fibrosis, mice were orally treated with three doses of silybin meglumine (50 mg/kg/d, 100 mg/kg/d, 200 mg/kg/d, Lot#: 2020102, Qianjin Pharmaceutical Co., Ltd., Zhuzhou, China.) [24] or tiopronin (20 mg/kg/d; Lot#: HY-B0373CS-2431; MedChemExpress, Monmouth Junction, NJ, USA) [25] from the first week, once a day for six weeks. The control and model groups received daily CMC-Na administration via gavage. Tiopronin, known for its capacity to protect hepatocytes by scavenging free radicals and promoting regeneration, was employed as a reference in this study [26]. The animals were euthanized 2 h after the final CCl_4_ administration. Blood, liver, bile, and intestinal content samples were collected.

### 2.2. LC-MS/MS

Mouse plasma (0.1 mL), liver tissue homogenate (20%, 100 μL), bile (100-fold dilution, 100 μL), and intestinal content homogenate (50%, 100 μL) were mixed with 10 μL of an internal standard solution (10 μg/mL), respectively. Subsequently, 1 mL of acetonitrile containing 5% ammonia water was added, followed by 3 min of vortexing. After centrifuging at 4000 rpm for 15 min, 900 µL of the supernatant was collected and evaporated in a vacuum concentrator at 45 °C for 4 h. The residue was then dissolved in 100 µL of a mobile phase (methanol:water = 85:15) and vortexed for 3 min. LC-MS/MS was performed using an Agilent (Santa Clara, CA, USA) Poroshell SB-C18 chromatographic column (150 × 3.0 mm, i.d. 2.7 μm) and TSQ Quantum Ultra Liquid Chromatography Mass Spectrometry (Thermo Scientific, Grand Island, NY, USA) at 35 °C. The mobile phases consisted of methanol (A) and a 5 mM ammonium acetate solution containing 0.1% formic acid (B). The flow rate was 0.1 mL/min, and 10 μL of the sample was injected. The HPLC gradient program started at 10% solvent A and 90% solvent B, shifted to various ratios, peaked at 95% A and 5% B, and returned to the initial 10% A and 90% B in 45 min. The concentration of BA stock solution was 0.1 mg/mL for β-muricholic acid (β-MCA) or 1.0 mg/mL for the other 21 types of BAs in chromatography-grade methanol.

### 2.3. Histological Analysis of Mouse Liver Tissue

Mouse liver tissue was fixed in 4% paraformaldehyde at 4 °C for 24 h. After fixation, the tissue was immersed in a 30% sucrose solution until sinking occurred. Following dehydration, the tissue was embedded in optimal cutting temperature compound and sectioned into 5 µm thick slices. The sections were stained with hematoxylin and eosin (H&E), picrosirius red (PSR), and Masson’s trichrome, as previously described [27,28].

### 2.4. Biochemical Analysis

Serum levels of alanine transaminase (ALT), aspartate transaminase (AST), gamma-glutamyl transferase (GGT), and total bile acid (TBA), as well as hepatic levels of hydroxyproline (HYP; A030-2-1), TBA, and malondialdehyde (MDA), were measured using ELISA kits from Nanjing Jiancheng Bioengineering Institute (Nanjing, China).

### 2.5. Integration of Silybin Meglumine–Bile Acid Disorder Targets

Silybin meglumine-related targets were obtained by using Pharmmapper database with “Silybin” 3D model from pubchem database as the keyword. These targets were converted into corresponding gene symbols using the UniProt database. Then, related targets were queried using the GeneCards database with “bile acid disorder” as the keyword, and the results were filtered to include targets with a GeneCards score greater than 10. These findings were then integrated with results from the Omim database. After removing duplicates, a list of gene symbols related to bile acid disorder was obtained. Finally, a silybin–bile acid disorder target map was created by finding the common targets between silybin and bile acid disorder. This intersection was visualized through a Venn diagram.

### 2.6. Protein–Protein Interaction (PPI) Network

A PPI network diagram was constructed to investigate the potential interactions and functional relevance of silybin–bile acid disorder targets. The intersecting targets were uploaded to the STRING platform, with a focus on the human species and a minimum interaction threshold set to highest confidence > 0.7.

### 2.7. Gene Enrichment Analysis

Gene ontology (GO) and Kyoto Encyclopedia of Genes and Genomes (KEGG) enrichment analyses were conducted using the Metascape platform, with a significance threshold of *p* < 0.01. The results were visualized using the Weishengxin online mapping platform (http://www.bioinformatics.com.cn/, accessed on 13 June 2024).

### 2.8. The qRT-PCR and Western Blot

Total RNA was isolated from liver tissue samples using Trizol (Invitrogen, Grand Island, NY, USA, 15596026) following the manufacturer’s instructions. The mRNA was reverse transcribed to cDNA templates using HiScript^®^ Q RT SuperMix for qPCR (+gDNA Wiper) reverse transcription kit (Vazyme, Nanjing, China, Q111-02/03) cDNA was synthesized using AceQ^®^ SYBR Green Master Mix Kit (High ROX Premixed). The qRT-PCR reactions were conducted using Applied Biosystems StepOne^TM^ real-time quantitative PCR. GAPDH was used as the reference gene. Relative expression levels were calculated using the 2^−ΔΔCt^ method. The primer sequences (Invitrogen, Grand Island, NY, USA) are summarized in Table 1.

We placed the liver tissue (50 mg) in SDS lysis buffer (Beyotime, Nanjing, China, P0013G) on ice for 30 min. The extraction of protein was then centrifuged at 12,000 rpm in lysis buffer for 15 min. Next, we measured the protein concentration using the BCA kit. Equal amounts of protein (120 μg) were then separated via sodium dodecyl sulfonate-polyacrylamide gel electrophoresis (SDS-PAGE) and transferred to a polyvinylidene difluoride (PVDF) membrane. PVDF membrane was placed in 5% nonfat dry milk for 2 h at room temperature and then incubated with primary antibody at 4 °C overnight. GADPH antibody (Santa Cruz Biotechnology, Dallas, TX, USA, sc-47724), *Collagen I* antibody (Abcam, USA, ab260043), and alpha-smooth muscle (Cell Signal Technology, Boston, MA, USA, 19,245 s) were all diluted in 1:1000 formula. The membranes were washed three times in TBST after incubation with primary and secondary antibodies, respectively. Protein bands were detected using an enhanced chemiluminescence detection kit (GlpBio, Montclair, CA, USA) and detected by the ChemiDoc XRS imaging system (Bio-Rad, Hercules, CA, USA).

### 2.9. Statistical Analysis

Data were presented as mean ± SEM. Statistical analysis was performed using a one-way ANOVA with GraphPad Prism 7.3.0 software. Each experiment was independently replicated at least three times. A *p* value less than 0.05 was considered statistically significant.

## 3. Results

### 3.1. CCl_4_ Induces Liver Fibrosis and Bile Acid Profile Alterations in a Mouse Model

To examine the therapeutic effect of SLB-M on liver fibrosis, we established a CCl_4_-induced liver fibrosis mouse model (Figure 1A). We utilized various staining techniques to evaluate tissue alterations. In the control group, histological integrity was maintained, as evidenced by H&E staining, with minimal collagen presence in PSR staining and typical collagen distribution in Masson’s trichrome staining. Conversely, the model group displayed disrupted histology in H&E, increased collagen expression as indicated by augmented brown staining in PSR, and enhanced blue staining in Masson’s trichrome, suggesting collagen deposition and fibrosis (Figure 1C,D). Furthermore, the model group exhibited significantly greater liver weight and elevated levels of HYP, ALT, and AST compared to the control group (Figure 1B). These findings suggest the successful replication of fibrotic changes and liver damage in this model.

To assess the impact of liver fibrosis on systemic and hepatic BA metabolism, we compared BA concentrations in blood and liver samples. The blood samples from the model group showed a marked elevation of all BA types. However, in the liver, while the levels of glycine-conjugated and taurine-conjugated BA remained unchanged, unconjugated BA levels significantly decreased when comparing the CCl_4_ group to the control group (Figure 1D). PCA revealed distinct clustering between the model and control groups, with individual BAs contributing differentially to the separation, indicative of altered BA metabolism in liver fibrosis. Specific BA concentrations, including β-MCA, cholic acid (CA), ω-muricholic acid (ω-MCA), glycocholic acid (GCA), and tauro-β-muricholic acid (T-β-MCA) concentrations, were notably elevated in the blood samples of the CCl_4_ group (Figure 1E), while in the liver, only β-MCA showed a statistically significant reduction (Figure 1F,G). These results suggest a more pronounced effect of CCl_4_ treatment on systemic BA metabolism than hepatic levels.

### 3.2. Silybin Meglumine Alleviates Liver Fibrosis in the Murine Model

**Figure 2:**
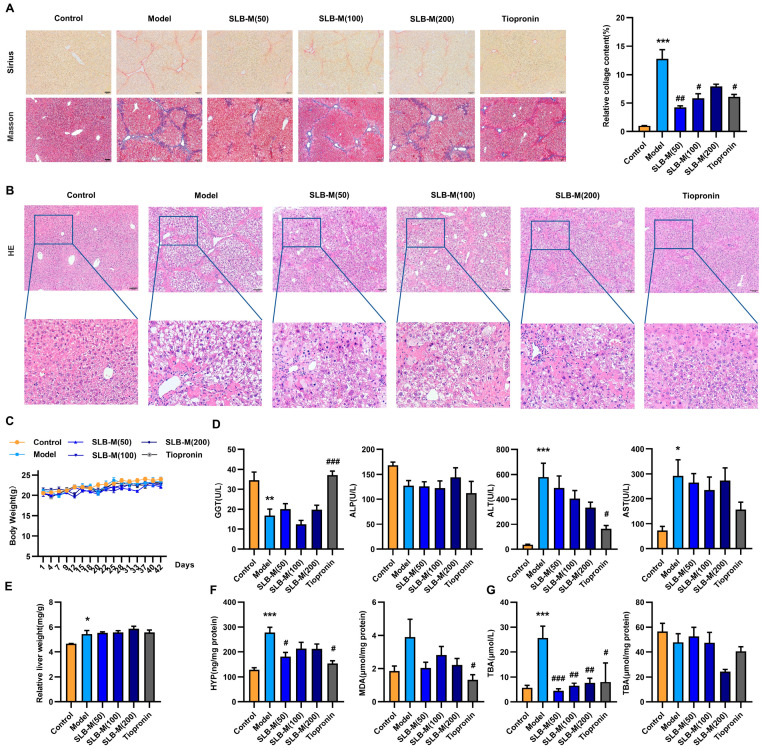
Therapeutic effects of silybin meglumine and tiopronin on CCl_4_-induced liver fibrosis in C57BL/6 mice. (**A**) Comparative histological outcomes, picrosirius red and Masson’s trichrome, to visualize collagen presence and fibrosis. Scale bar = 100 µm. Relative collagen content of Masson staining. Data represent at least three independent experiments with triplicate measurements. (**B**) Liver sections were stained with hematoxylin and eosin (HE) to visualize structural changes. (**C**) Body weight trajectories of mice over the study period. (**D**) Serum levels of alanine aminotransferase (ALT), aspartate aminotransferase (AST), gamma-glutamyl transferase (GGT), alkaline phosphatase (ALP). (**E**) Quantification of liver weight to body weight ratio. (**F**,**G**) Liver tissue levels of hydroxyproline (HYP), malondialdehyde (MDA), total bile acids (TBA), and blood levels of TBA. Data are represented as mean ± SEM; * *p* < 0.05, ** *p* < 0.01, *** *p* < 0.001 vs. control; ^#^ *p* < 0.05, ^##^ *p* < 0.01, ^###^ *p* < 0.001 vs. model; *n* = 6.

To investigate the effect of SLB-M on liver fibrosis, we treated model mice with SLB-M or tiopronin for two weeks from the beginning of the modeling. An experimental design schematic allowed for the evaluation of silybin meglumine and tiopronin treatment on CCl4-induced liver fibrosis in mice. CCl4-induced mice were treated with high-dose silybin meglumine (SLB-M, 200 mg/kg/d), medium-dose silybin meglumine (SLB-M, 100 mg/kg/d), low-dose silybin meglumine (SLB-M, 50 mg/kg/d), or tiopronin (20 mg/kg/d). Histological analysis showed tissue restoration and reduced collagen deposition in silybin meglumine- or tiopronin-treated mice compared to the untreated model mice (Figure 2A,B). No significant differences were observed in body weights or liver weight to the body weight ratios in the silybin meglumine- and tiopronin-treated groups compared to the model group (Figure 2C,E). Significantly elevated serum levels of ALT, AST, and TBA were observed in the model group, along with a marked decrease in GGT. These changes, accompanied by significantly increased hepatic levels of HYP, were effectively reversed by SLB-M treatment, suggesting its hepatoprotective and antifibrotic potential (Figure 2D,F,G). It appears that 50 mg/kg/day was the lowest effective dosage, which was applied in the subsequent analysis.

### 3.3. Silybin Meglumine Modulates BA Composition in CCl_4_-Induced Liver Fibrosis Mouse Model

**Figure 3:**
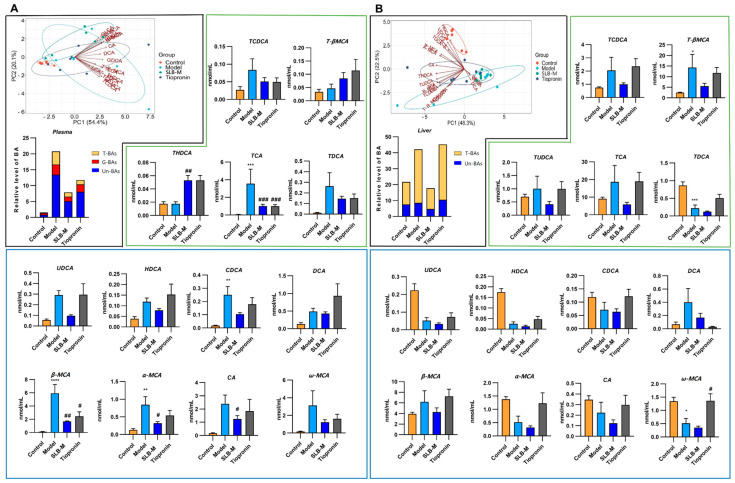
PCA plots and bar graphs from LC-MS/MS analysis demonstrated the modulation of BA profiles by silybin meglumine treatment in the plasma (**A**) and liver (**B**) of control, model and silybin meglumine- and tiopronin-treated mice. Data are represented as mean ± SEM; * *p* < 0.05, ** *p* < 0.01, *** *p* < 0.001, **** *p* < 0.0001 vs. control; **^#^** *p* < 0.05, **^##^** *p* < 0.01, **^###^** *p* < 0.001 vs. model; *n* = 6.

To investigate the effects of SLB-M on BA metabolism in liver fibrosis, we conducted PCA and LC-MS/MS analysis. PCA revealed a distinct separation among the four groups in all biological samples (Figure 3 and Figures S1–S3). In the blood sample PCA biplot, a modest separation between the model (blue) and SLB-M-treated (green) groups was observed (Figure 3A, left). In the liver and bile samples, the separation occurred prominently along PC2 (Figure 3B (left) and Figure S1). Similarly, in the ileum and cecum contents, a separation between the two groups was also noted (Figures S2 and S3). Furthermore, a tighter clustering and smaller ellipse area were observed for the SLB-M-treated group compared to the model group across most samples, indicating effective stabilization or regulation of BA levels in response to silybin meglumine treatment.

Moreover, the results of the LC-MS/MS analysis indicated a significant increase in all categories of BAs in the plasma of the model group compared to the control, which was effectively attenuated by both SLB-M and tiopronin treatments (Figure 3A, left). SLB-M treatment also demonstrated a similar effect in the liver (Figure 3B, left) but not in the ileum contents (Figure S2). Conversely, the model group exhibited a decrease in taurine-conjugated BAs in the bile and unconjugated BAs in the cecum contents. Both SLB-M and tiopronin treatments reversed this decrease, with tiopronin showing a more pronounced effect (Figures S1 and S3). Specifically, in the plasma, β-muricholic acid (β-MCA), alpha-muricholic acid (α-MCA), and glycolithocholic acid (GLCA) were significantly elevated in the model group compared to the control, with SLB-M treatment reversing this effect. Similar trends were observed in other BAs such as cholic acid (CA), omega-muricholic acid (ω-MCA), and taurocholic acid (TCA) (Figure 3A). Comparable trends were also observed in the liver, albeit nonsignificant (Figure 3B). Additionally, the SLB-M exhibited a reversal effect on some BAs like B-MCA and TCA in the bile (Figure S1) as well as α-MCA and ω-MCA in the cecum contents despite its nonsignificance in some instances (Figures S2 and S3). Taken together, these results suggest that SLB-M treatment effectively attenuates dysregulation of BA metabolism induced by liver fibrosis.

### 3.4. Identification and Characterization of DEGs in Silybin Meglumine-Treated Liver Fibrosis

**Figure 4:**
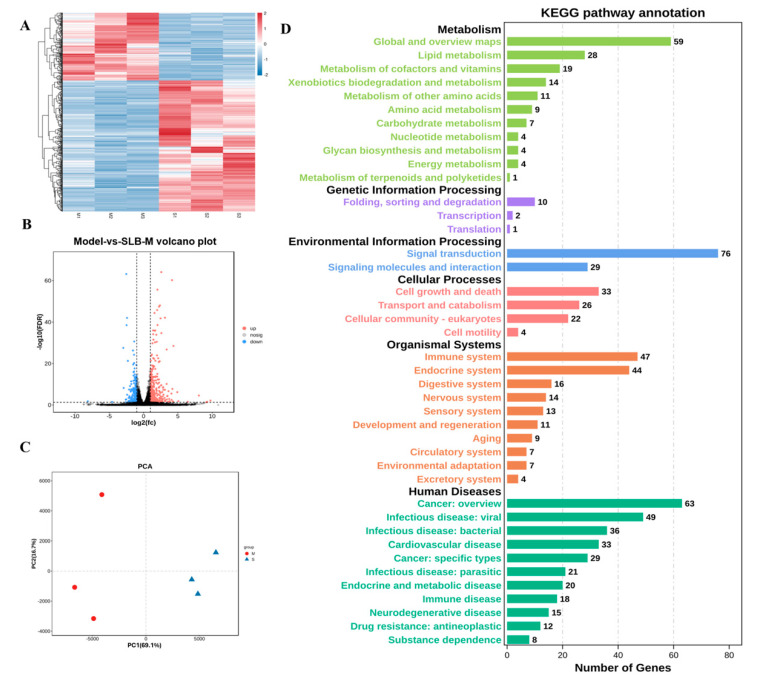
Identification of differentially expressed genes (DEGs) in liver tissues from silybin meglumine-treated mice (S1–S3) and untreated model mice (M1–M3). (**A**) Heatmap with hierarchical clustering depicts gene expression levels across samples. (**B**) A PCA scatter plot demonstrates the separation between the two groups. (**C**) A volcano plot identifies DEGs based on log2 fold-change and adjusted *p*-value. (**D**) KEGG pathway analysis indicates the predominant involvement of DEGs in various pathways.

To further explore the potential therapeutic mechanism of SLB-M on liver fibrosis, we collected liver tissue samples from untreated and treated model mice (*n* = 3/group). Hierarchical clustering stratified samples by gene expression patterns (Figure 4A). PCA showed clear separation between the groups (Figure 4B). A total of 502 DEGs were identified, with 331 upregulated and 171 downregulated in the SLB-M-treated group (Figure 4C). The bubble plot illustrates pathway enrichment significance. The top 20 impacted pathways included the PPAR signaling pathway involved in the protective response against liver fibrosis [29] and the Hippo signaling pathway associated with the regulation of BA metabolism [29,30] (Figure S4). KEGG pathway analysis showed that these DEGs were primarily engaged in metabolic pathways and signal transduction (Figure 4D).

### 3.5. Network Pharmacology Analysis of Potential Therapeutic Mechanisms of Silybin Meglumine in Liver Fibrosis

**Figure 5:**
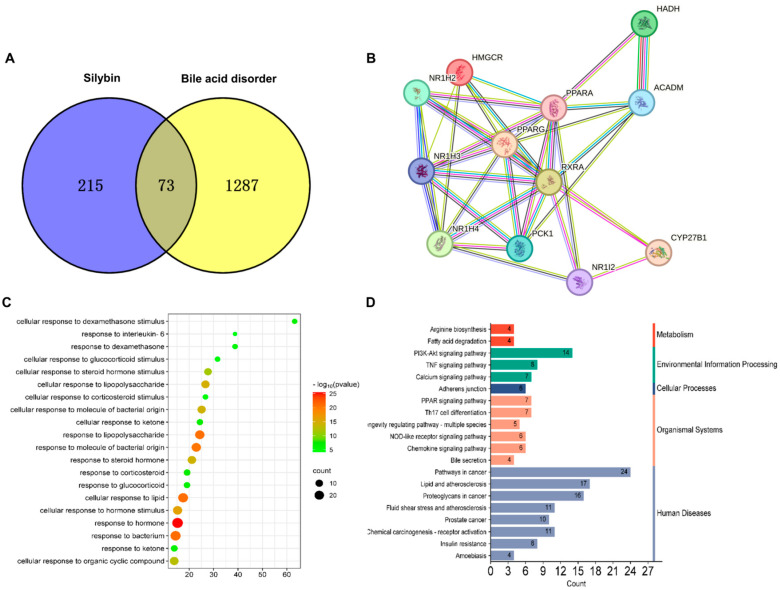
Bioinformatics analysis of silybin meglumine’s therapeutic mechanism. (**A**) Venn diagram displaying the overlap of silybin targets and genes associated with bile acid disorder. (**B**) Protein–protein interaction (PPI) network for the 12 intersecting genes. (**C**) Gene ontology (GO) analysis outcomes. (**D**) Kyoto Encyclopedia of Genes and Genomes (KEGG) pathway analysis results.

To understand the potential therapeutic mechanisms of SLB-M in liver fibrosis, we employed comprehensive bioinformatics analysis. We identified a total of 288 silybin targets through the Pharmmapper platform. Additionally, we found 1360 genes associated with bile acid disorder from the GeneCards database. A Venn diagram revealed that 73 genes were common between silybin and bile acid disorder (Figure 5A and Table S1). A PPI network analysis suggested the most potential interactions and functional relevance among these genes (Figure 5B). A GO analysis showed that these genes were predominantly involved in biological processes related to a cellular response to stimulus, with a marked significance in pathways such as steroid hormone, cellular response to lipid, and organic cyclic compound (Figure 5C). The GO terms were significantly associated with critical signaling and regulatory mechanisms, including IL-6 signaling. A KEGG pathway analysis implicated these targets predominantly in pathways related to cancer, metabolism, and bile secretion (Figure 5D).

### 3.6. Silybin Meglumine Modulates Bile Secretion Gene Expression through the Activation of Fxr in Liver Fibrosis

**Figure 6:**
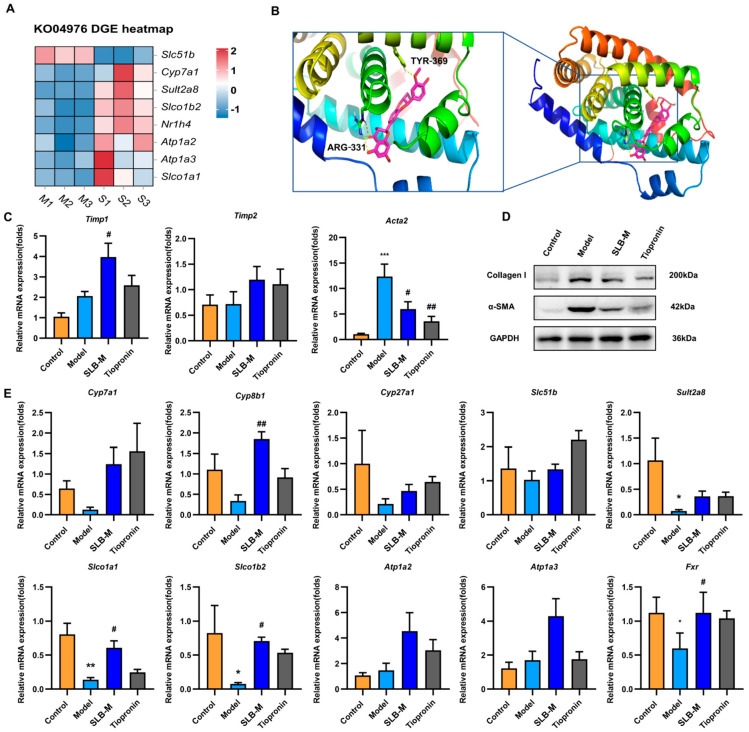
Differential gene expression analysis related to bile secretion and validation of the impact of silybin meglumine on gene expression in liver fibrosis and bile secretion. (**A**) A heatmap displays differential gene expression in the bile secretion pathway (ko04976) in silybin meglumine-treated (S1, S2, S3) versus model group samples (M1, M2, M3) (*n* = 3). Schematic representation of the bile secretion pathway with emphasis on *OATPs*, *Cyp7a1*, *Fxr*, *Sult2a1*, and *OST-β*. (**B**) A 3D visualization of the protein–ligand complex demonstrates the spatial conformation of the secondary structures of *Fxr* around silybin. (**C**) A qRT-PCR was performed to validate the impact of SLB-M on gene expression in liver fibrosis from control, model and SLB-M- and tiopronin-treated mice. (**D**) The protein expression of *α-SMA* and *Col1a1* in liver tissue, detected by Western blot. *GAPDH* was used as a reference control for equal protein loading. (**E**) A qRT-PCR was performed to validate the impact of SLB-M on gene expression in bile secretion from control, model and SLB-M- and tiopronin-treated mice liver tissue. Data are represented as mean ± SEM; * *p* < 0.05, ** *p* < 0.01, *** *p* < 0.001 vs. control; ^#^ *p* < 0.05, ^##^ *p* < 0.01 vs. model; *n* = 6.

To investigate the potential mechanism underlying SLB-M’s modulation of bile secretion, a differential gene expression analysis was conducted between the untreated and treated model mice using the KEGG pathway identifier ko04976 for “Bile secretion”. This analysis identified key genes with altered expression profiles and separated the samples into two distinct clusters. Specifically, *Slc51b* (*OSTβ*) was downregulated, while *Cyp7a1*, *Sult2a8*, and others were upregulated in the SLB-M-treated group. In the bile secretion pathway, genes like *OATPS*, *Cyp7a1*, *Fxr*, *Sult2a1,* and *OSTβ* were highlighted (Figure 6A). It is known Farnesoid X receptor (*Fxr*) exhibiting antifibrotic properties by inhibiting HSC activation and proliferation, which are central to the fibrotic process [31]. *Fxr* also regulates genes involved in BA secretion [31]. A ligand–receptor interaction analysis revealed that silybin may competitively occupy the retinoic acid binding site of *Fxr* (Figure 6B). We also quantitatively measured the binding mode and binding energies of the *Fxr* agonist obeticholic acid (OCA) to *Fxr* and tiopronin to *Fxr*. OCA forms four hydrogen bonds in *Fxr* while tiopronin and SLB-M have two bonds. The free binding energy is −81.152 kcal kcal/mol (OCA) and −32.18 kcal/mol (tiopronin), while silybin is −58 kcal/mol (Figure S5). Although its binding energy is lower than OCA’s, it still exhibits a certain degree of stability and binding affinity. Moreover, our results demonstrated that SLB-M potentially contributes to increasing the transcriptional level of *Fxr,* indicating its mechanism may be different from a direct agonist on *Fxr*. These data suggest that SLB-M treatment modulates the expression of genes critical for BA synthesis and transport.

To verify the impact of SLB-M on liver fibrosis and bile secretion, we conducted a qRT-PCR and Western blot to measure the expression levels of markers related to these processes in mouse liver samples. The data revealed that SLB-M counteracted the upregulation of *Acta2* and the downregulation of genes related to bile secretion such as *Slco1a1*, *Slco1b1*, and *Sult2a8* observed in the model mice (Figure 6C,E). Hence, to further verify the role of SLB-M in reversing fibrosis, the protein expression alteration markers upon fibrosis treatment were measured by Western blot. The results revealed a downregulation in *α-SMA* and *Collagen I* expression in the liver tissue of SLB-M-treated mice, indicating that SLB-M has protective effects on the liver, which is consistent with our findings from the PCR experiment (Figure 6D). These data suggest that SLB-M modulates gene and protein expression related to liver fibrosis and bile secretion, underscoring its therapeutic potential.

## 4. Discussion

Silybin, primarily known for its antioxidant, anti-inflammatory, and liver-protective effects, is widely used in treating various liver diseases including chronic liver diseases, cirrhosis, and hepatocellular carcinoma [32]. However, research on its effects on BA metabolism is limited, highlighting a need for further exploration in this area. In this study, we found that SLB-M treatment ameliorated liver fibrosis by reducing collagen deposition and restoring tissue integrity by promoting the *Fxr* to appropriately decrease conjugated BAs by promoting *Slco1a1*, *Slco1b1*, and *Sult2a8* expression. Additionally, SLB-M reversed alterations in BA metabolism induced by CCl_4_, normalizing BA composition in various biological matrices. Mechanistically, SLB-M modulated the expression of genes critical for BA transport, indicating its role in regulating liver fibrosis and bile secretion pathways. These findings highlight the therapeutic efficacy of SLB-M in liver fibrosis and its potential as a treatment for related metabolic disorders.

Through the assessment of hepatic enzymes and markers indicative of liver damage, we found that the CCl_4_-induced model group exhibited significantly elevated serum levels of ALT, AST, and TBA, which are classical indicators of hepatocellular injury and cholestasis [33]. Interestingly, GGT levels were decreased in the model group, a deviation from typical cholestatic profiles where GGT is often elevated [34]. This atypical finding might suggest a differential response to CCl_4_-induced liver injury or a possible compensatory mechanism in hepatic pathology. Furthermore, the model group showed a significant increase in hepatic HYP, a marker of collagen content and fibrosis [35], and MDA, a marker of lipid peroxidation and oxidative stress [36]. However, no significant alterations in liver TBA were observed, suggesting that the cholestatic effect observed in serum did not correspond to a similar change in the liver tissue. This could be reflective of the early stage or specific type of fibrotic pathology that does not uniformly affect all markers or may indicate localized hepatic damage that has not uniformly affected the entire liver. The administration of SLB-M and tiopronin significantly reversed these biochemical indicators, supporting their potential role in hepatoprotection and antifibrotic therapy.

Based on the results of PCA and LC-MS/MS analysis, we found that SLB-M treatment effectively attenuated dysregulation of BA metabolism induced by liver fibrosis, as evidenced by significant reductions in plasma BA levels and reversal of alterations in BA composition in various biological samples. BAs are synthesized from cholesterol in the liver, conjugated with amino acids to increase solubility, and then secreted into bile. BAs are stored in the gallbladder and released into the duodenum to aid fat digestion. Most BAs are reabsorbed in the ileum and returned to the liver. Some are transformed by gut microbiota in the colon into secondary forms, either reabsorbed or excreted in feces. A small amount enters the bloodstream and is excreted in urine [37]. When liver fibrosis occurs, the liver tissue becomes progressively scarred due to continuous damage and inflammation [38]. Liver fibrosis can cause cholestasis by blocking bile ducts with fibrotic scarring, disrupting the enterohepatic circulation of BAs and leading to BA accumulation in the liver and increased levels in plasma, bile, and feces [39]. Furthermore, liver damage can disrupt the expression and function of transporters responsible for BA uptake and secretion [40], leading to reduced hepatic retention and an increased systemic release of BAs. Consistent with our findings, in patients with non-alcoholic fatty liver disease, particularly those with worsening fibrosis, there were notable elevations in BA concentrations both in serum and fecal samples compared to healthy controls. Specifically, CA and LCA were significantly elevated in patients with worsening fibrosis. Similarly, among the fecal BA fraction, CA, DCA, and LCA were notably elevated [41]. Our study demonstrates that SLB-M treatment not only reduces the elevated levels of BAs in various biological samples but also corrects the altered BA composition in liver fibrosis, potentially by improving liver function and normalizing the expression and activity of BA transporters disrupted by fibrosis.

Therefore, we further explored the impact of SLB-M on gene expression related to bile secretion. Differential gene expression analysis revealed key alterations in genes involved in the bile secretion pathway. Genes such as *Slc51b* were downregulated, while *Slco1a1*, *Slco1b1*, *Sult2a8*, and others were upregulated in SLB-M-treated mice. This was further corroborated by qRT-PCR data showing that SLB-M reversed the alterations in fibrosis-related and bile secretory gene expression, including the modulation of genes like *OATPs*, *Fxr*, and *Sult2a8*. *SLC51B* (solute carrier family 51, beta subunit), also known as the organic solute transporter beta (*OSTβ*), forms a heteromeric complex with *OSTα* (*SLC51A*) to function as an organic solute transporter. This transporter is primarily located in the ileum of the small intestine and the liver, facilitating the efflux of BAs from enterocytes into the portal circulation and from hepatocytes into bile [42]. *Cyp7a1* is the key enzyme in BA synthesis from cholesterol, initiating the conversion of cholesterol into primary BAs in the liver [43]. *Sult2a8* (sulfotransferase family 2a member 8) plays a crucial role in the sulfation of BAs, contributing to BA metabolism and regulation in biological systems [44]. Alterations in the expression or function of these genes have been reported to significantly impact BA transport and homeostasis in liver fibrosis [45,46,47]. Western blot results revealed a downregulation in *α-SMA* and *Collagen I* expression in the liver tissue of SLB-M-treated mice, indicating that SLB-M has been shown to alleviate hepatic injury by modulating the expression of genes and proteins involved in BA homeostasis [48], consistent with our findings. Based on our research, SLB-M may ultimately lead to a decrease in bile acid content through the enhancement of bile acid transporter activity such as *Slco1a1*, *Slco1b1*, and *Sult2a8*. Literally, the expression of *Cyp7a1* and *Cyp27a1* was increased based on RNA-seq and RT-PCR results, but this is inconsistent with the role of SLB-M in reducing liver TBA. Considering the increase in bile acid, biosynthesis may be a negative feedback during the process of increased bile acid efflux.

Bile acids transport primarily relies on the form of conjugated bile acids. The decrease of conjugated acids in the liver was based on the protective effects SLB-M, which raised the expression of *Slco1a1*, *Slco1b1*, and *Sult2a8*. Conjugated bile acids are generally less toxic than unconjugated bile acids, but the mechanism of them in SLB-M is still unclear and requires further investigation.

The primary limitation of this study is its reliance on a murine model, which may not fully replicate the pathophysiology of human liver fibrosis. Further research involving diverse animal models and clinical trials is needed to validate these findings and to understand the broader applicability and mechanisms of SLB-M in treating liver fibrosis.

In conclusion, this study demonstrates that SLB-M significantly mitigates liver fibrosis and restores BA homeostasis in a CCl_4_-induced murine model. Additionally, it reveals SLB-M’s impact on the expression of key genes such as *Slco1a1, Slco1b1, Sult2a8, Fxr, and OSTβ,* which we believe contribute to converting cholesterol into BAs. Further results concluded that SLB-M increased unconjugated BAs like cholic acid and chenodeoxycholic acid but decreased conjugated BAs including taurocholic acid and taurodeoxycholic acid. The potential mechanism might be mainly attributed to *Fxr* activation by altering BA composition. Although our present study underscores SLB-M’s potential as a multi-targeted therapeutic agent for liver fibrosis with implications for both liver tissue repair and the regulation of BA pathways, the relationship among SLB-M, the liver–gut microbiota axis, and liver fibrosis is still unclear and needs to be confirmed by further studies.

## Figures and Tables

**Table 1:** RT-PCR primer sequence.

Gene	Forward (5′–3′)	Reverse (5′–3′)
*Acta2*	GTACCACCATGTACCCAGGC	GCTGGAAGGTAGACAGCGAA
*Atp1a2*	ACAGGAACCCTAAGGTGGCAGA	GTGGCTGAACTTGAGGAAACGG
*Atp1a3*	GGTGTGGGTATCATCTCTGAGG	CGTCAATCTGCTCCGAGGTGAA
*Cyp27a1*	GGAAGGTGCCCCAGAACAA	GCGCAGGGTCTCCTTAATCA
*Cyp7a1*	CAGGGAGATGCTCTGTGTTCA	AGGCATACATCCCTTCCGTGA
*Cyp8b1*	GTACACATGGACCCCGACATC	GGGTGCCATCAGGGTTGAG
*GADPH*	ATGGAGAAGGCTGGGGCTCACCT	AGCCCTTCCACGATGCCAAAGTTGT
*FXR*	TGGACTCATACAGCAAACAGAGA	GTCTGAAACCCTGGAAGTCTTTT
*Slc51b*	CAAGCATGTTCCTCCTGAGAAGG	CTCTTAGGAAGACCTGGCTGTTG
*Sult2a8*	AAACAGCAAGGAGGGTCCACGT	CCTGACACAAGAACATCTCTGGG
*Slco1b2*	GCAATGATCGGACCAATCCTTGG	CCAACGAGCATCCTGAGGAGTT
*Slco1a1*	GCTGTTCAGTCTTACGAGTGTGC	CAAGGCATACTGGAGGCAAGCT
*Timp1*	AGATACCATGATGGCCCCCT	CGCTGGTATAAGGTGGTCTCG
*Timp2*	CTGGGACACGCTTAGCATCA	CCATCCAGAGGCACTCATCC

## Data Availability

All data generated or analyzed during this study are included in this article and Supplementary Information files.

## Supplementary Materials

The following supporting information can be downloaded at https://www.mdpi.com/article/10.3390/metabo14100556/s1: Figure S1. PCA and LC-MS/MS analysis of BA profiles in bile of mice. Figure S2. PCA and LC-MS/MS analysis of BA profiles in ileum content of mice. Figure S3. PCA and LC-MS/MS analysis of BA profiles in cecum content of mice. Figure S4. GO analysis reveals enriched terms for the DEGs in three categories: biological processes (BPs), molecular functions (MFs), and cellular components (CCs). Left: a bubble plot illustrates the statistical significance of pathway enrichment among the DEGs. Right: summary of the top 20 pathways significantly impacted. Figure S5: (A) A 3D visualization of the protein–ligand complex demonstrates the spatial conformation of the secondary structures of *Fxr* around OCA. (B) A 3D visualization of the protein–ligand complex demonstrates the spatial conformation of the secondary structures of *Fxr* around tiopronin. Table S1. Intersection of silybin meglumine and bile acid disorder targets.
