# Supplementary material for: Silybin Meglumine Mitigates CCl4-Induced Liver Fibrosis and Bile Acid Metabolism Alterations
Source: Metabolites. 2024 Oct 17;14(10):556. doi: 10.3390/metabo14100556 (PMC11509150; doi:10.3390/metabo14100556)
Supplement: Supplementary file 1 [file metabolites-14-00556-s001.zip › Table S1.pdf]

Table S1. Intersection of silybin meglumine and bile acid disorder targets.

Serial number	Target name
1	ACADM
2	ADH1C
3	ADK
4	AKT1
5	ALB
6	ALDH2
7	ARG1
8	BCHE
9	BMP7
10	CASP3
11	CBS
12	CCL5
13	CYP2C9
14	DPP4
15	EGFR
16	ELANE
17	ESR1
18	F2
19	FABP3
20	FECH

21	FGFR1
22	GCK
23	GSTP1
24	HADH
25	HMGCR
26	HSP90AA1
27	HSPA8
28	IGF1
29	IL2
30	JAK2
31	KDR
32	KIT
33	LCN2
34	LGALS3
35	MAPK14
36	MAPK8
37	MDM2
38	MET
39	MIF
40	MMP2
41	MMP3
42	MMP7

43	MMP9
44	NMNAT1
45	NOS2
46	NOS3
47	NR1H2
48	NR1H3
49	NR1H4
50	NR1I2
51	OTC
52	PCK1
53	PLAU
54	PPARA
55	PPARG
56	PRKACA
57	PYGL
58	RBP4
59	REN
60	RHOA
61	RNASE3
62	RXRA
63	S100A9
64	SERPINA1

65	SOD2
66	SRC
67	TGFBR1
68	TTR
69	VDR
70	XIAP
71	YARS1
72	TYMP
73	TYMS
