# Supplementary figures and images for: Silybin Meglumine Mitigates CCl4-Induced Liver Fibrosis and Bile Acid Metabolism Alterations
Source: Metabolites. 2024 Oct 17;14(10):556. doi: 10.3390/metabo14100556 (PMC11509150; doi:10.3390/metabo14100556)

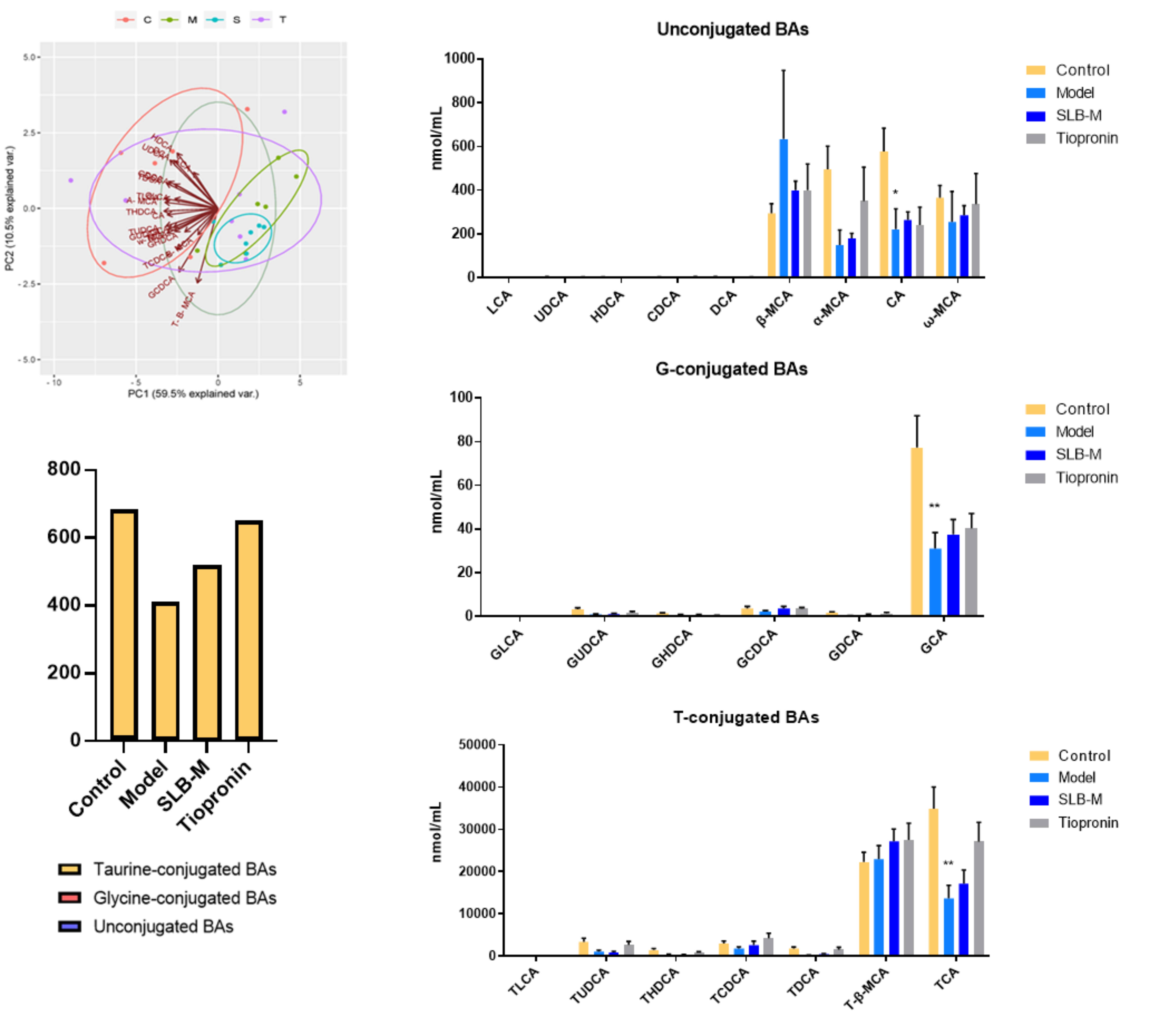

Supplement: Supplementary file 1 [file metabolites-14-00556-s001.zip › Figure S1.jpg]

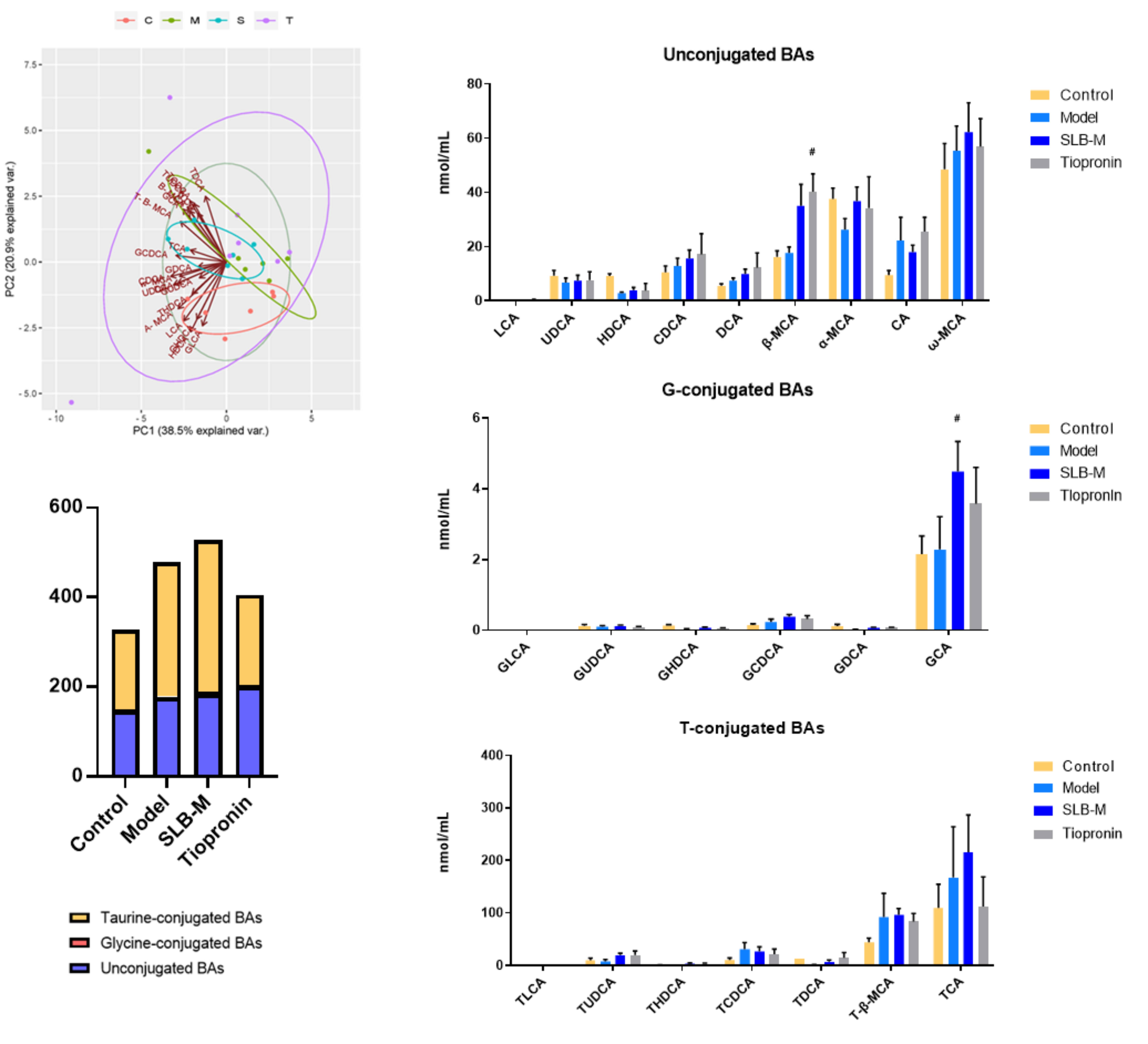

Supplement: Supplementary file 1 [file metabolites-14-00556-s001.zip › Figure S2.jpg]

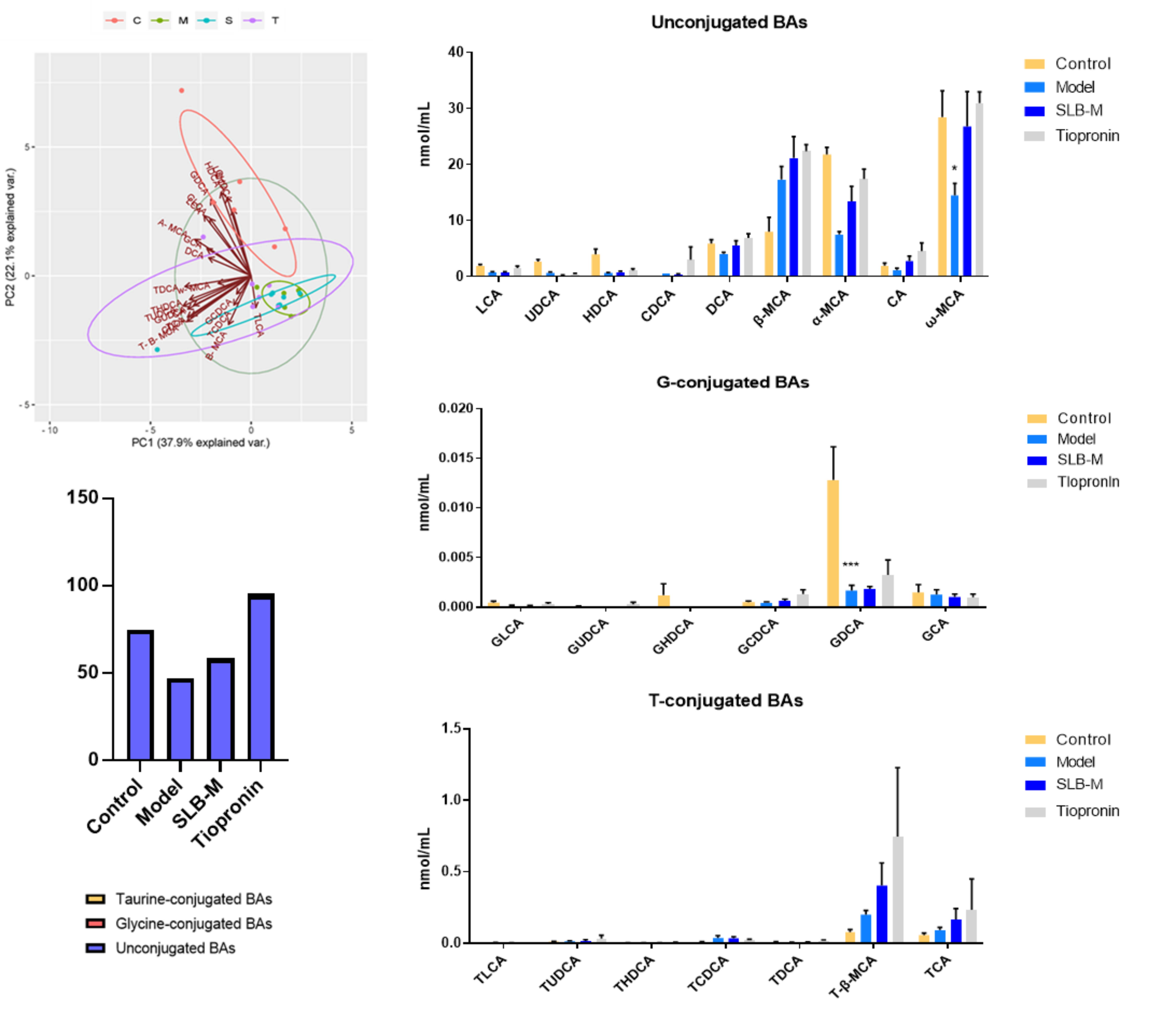

Supplement: Supplementary file 1 [file metabolites-14-00556-s001.zip › Figure S3.jpg]

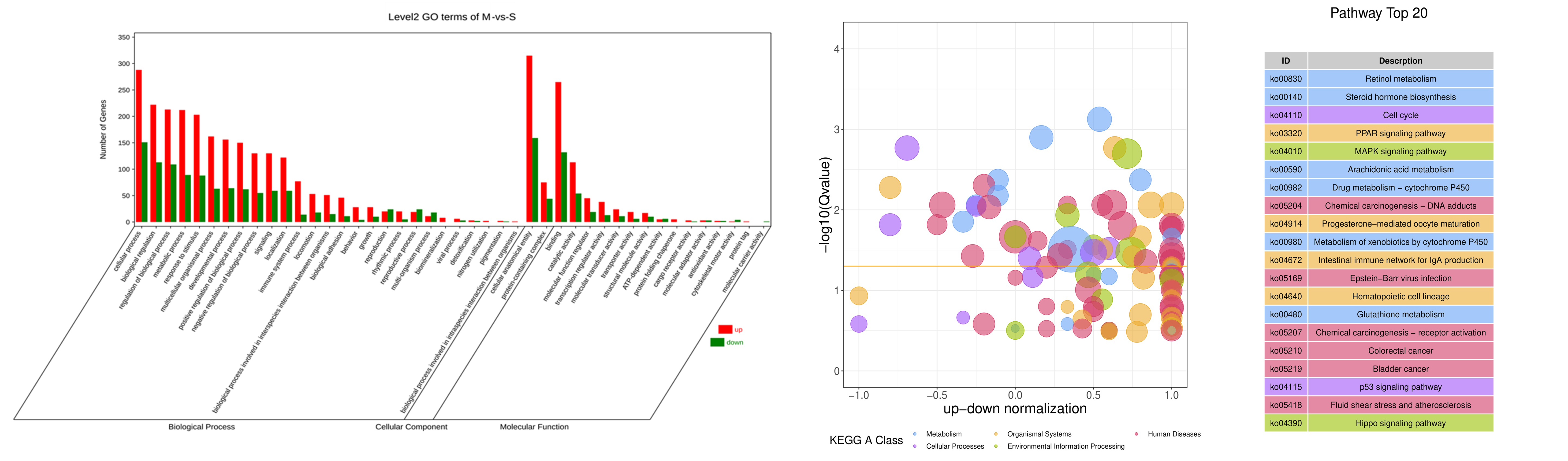

Supplement: Supplementary file 1 [file metabolites-14-00556-s001.zip › Figure S4.jpg]

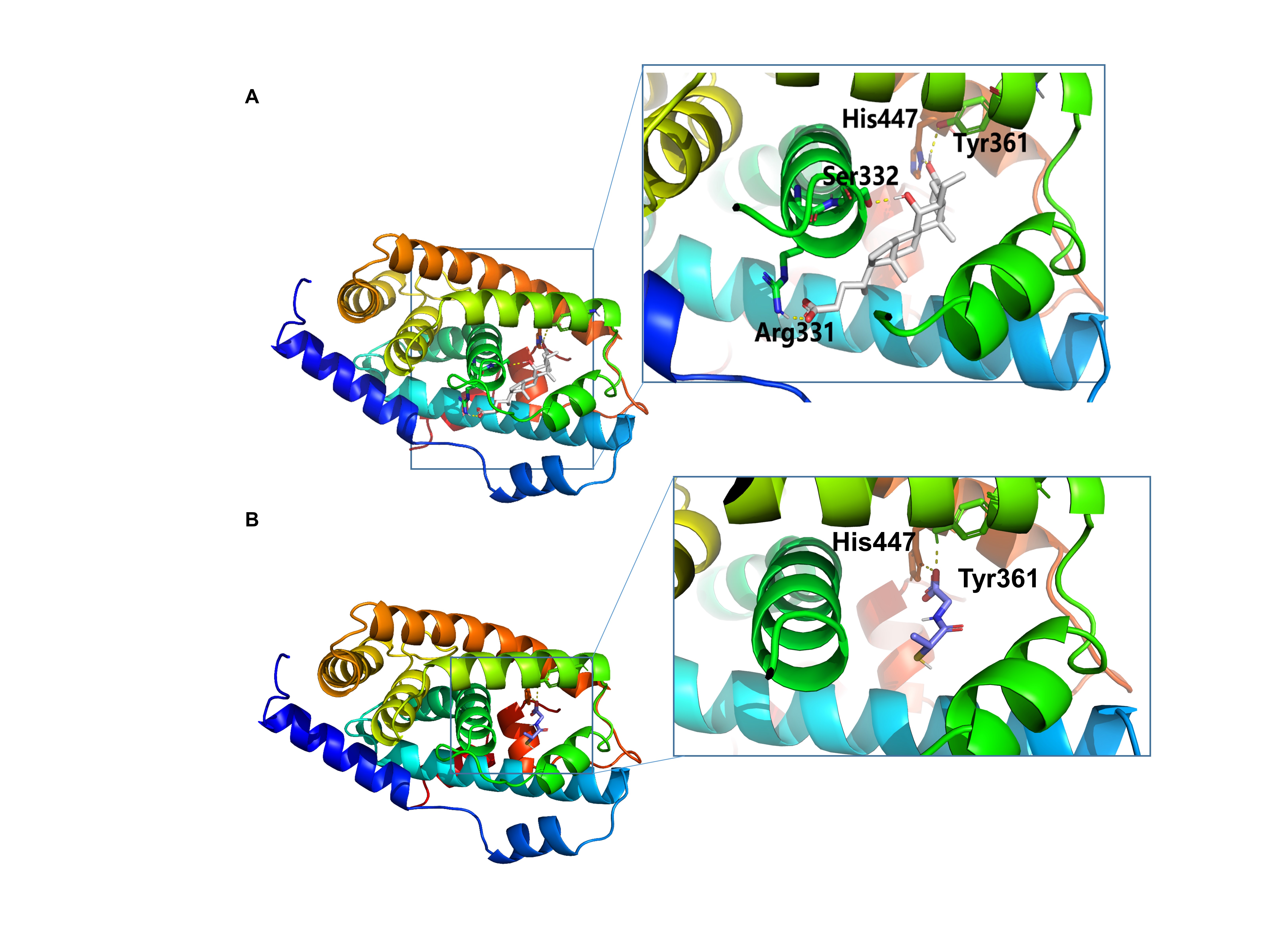

Supplement: Supplementary file 1 [file metabolites-14-00556-s001.zip › Figure S5.jpg]
